# Optical Tissue Clearing: Illuminating Brain Function and Dysfunction

**DOI:** 10.7150/thno.53979

**Published:** 2021-01-01

**Authors:** Xiaohan Liang, Haiming Luo

**Affiliations:** 1 Britton Chance Center for Biomedical Photonics, Wuhan National Laboratory for Optoelectronics-Huazhong University of Science and Technology, 430074, Wuhan, Hubei, China; 2 MoE Key Laboratory for Biomedical Photonics, Collaborative Innovation Center for Biomedical Engineering, School of Engineering Sciences, Huazhong University of Science and Technology, 430074, Wuhan, Hubei, China

**Keywords:** Optical tissue clearing, Neural circuits, Brain atlas, Brain diseases, Neurodevelopment, Image analysis tools

## Abstract

Tissue optical clearing technology has been developing rapidly in the past decade due to advances in microscopy equipment and various labeling techniques. Consistent modification of primary methods for optical tissue transparency has allowed observation of the whole mouse body at single-cell resolution or thick tissue slices at the nanoscale level, with the final aim to make intact primate and human brains or thick human brain tissues optically transparent. Optical clearance combined with flexible large-volume tissue labeling technology can not only preserve the anatomical structure but also visualize multiple molecular information from intact samples in situ. It also provides a new strategy for studying complex tissues, which is of great significance for deciphering the functional structure of healthy brains and the mechanisms of neurological pathologies. In this review, we briefly introduce the existing optical clearing technology and discuss its application in deciphering connection and structure, brain development, and brain diseases. Besides, we discuss the standard computational analysis tools for large-scale imaging dataset processing and information extraction. In general, we hope that this review will provide a valuable reference for researchers who intend to use optical clearing technology in studying the brain.

## Introduction

Imaging is an effective strategy to visually understand the structural and functional basis of organisms and the processes of various physiological activities and pathological mechanisms. Some commonly used biomedical imaging techniques, such as magnetic resonance imaging (MRI), computed tomography (CT), and positron emission tomography (PET), can image the anatomical details or metabolic processes. However, it is difficult to obtain information at the cellular level. Since the cell is the basic unit of structure and function of the organism, and different cell populations play a vital role in developing anatomical and functional circuits of an organism, novel imaging techniques with the single-cell resolution are urgently needed for comprehensive observation and analysis of organs or even the whole body. One possible approach is to expand classical histological methods that can only image thin tissue sections (at the level of 10 μm). Carrying out proper serial slice sectioning and slice reconstruction procedures may be an excellent way to visualize the three-dimensional organization of biological samples. However, the time-consuming nature and inevitable errors from tissue damage (tear, folding, compression, stretching, etc.) and reconstruction pipeline limit the broad application of this method. The optical clearing is a promising technique since deep fluorescent microscopic imaging of cleared samples may provide three-dimensional data with high spatial resolution 1. Due to the scattering properties of tissues, many techniques such as light-sheet, two-photon, multiphoton, confocal microscopy have limited imaging depths. Optical wavefront engineering is a possible method to correct light wavefront distortions caused by tissue heterogeneity to increase the imaging depth and improve cellular resolution 1. Optical clearing of tissues proposes another idea to enhance tissue uniformity, provides practical solutions to the above problems, and has broad application prospects in visualizing cross cell and organic layers 2.

When light irradiates biological tissues, it is absorbed and scattered, with light scattering being the main factor leading to the opacity of mammalian tissues 3. Biological tissue is mainly composed of water (refractive index, RI ~ 1.33) and a large number of scattering particles (such as protein, lipid, etc.) with a high refractive index (RI ~1.50) 4. The refractive index mismatch causes light to propagate at different speeds and angles when passing through biological tissues—multiple scattering, which eventually results in opaque biological samples. From this perspective, it would be better to match the refractive index of different tissue components by replacing low refractive index material (water) and removing the high refractive index materials (such as lipids). Finally, optical clearing procedures can improve tissue heterogeneity, reduce tissue scattering, increase the depth of light penetration in tissues, making it transparent.

It has been a century since the optical clearing of large tissues was first proposed 5. At present, tissue optical clearing technology has achieved intact mouse body imaging at subcellular-resolution 6-8. It has shown broad application potential in a three-dimensional reconstruction of tissue structure with high resolution, revealing pathogenic mechanism, illustrating pathological manifestations of various diseases, as well as drug screening. In this review, we will discuss the latest progress in the development and applications of tissue optical clearing technology from the perspective of technical users. We first describe the characteristics of conventional visual clearing methods and point out the factors that should be considered when choosing a suitable optical clearing solution. Afterwards, we will focus on the applications of optical clearing technology in brain research (brain atlas, brain development, brain diseases, etc.). Finally, we will summarize the existing quantitative analysis tools for optical clearing imaging, which will provide a valuable reference for researchers.

## Tissue optical clearing methods

Tissue optical clearing technology originated one hundred years ago was first performed using organic solvent 5. In the last decade, the great potential of the optical clearing method in biology research has been discovered due to gradual advances made in optical imaging technology. So far, the existing clearing methods can be classified into four categories: solvent, simple immersion (high RI aqueous solution), hyperhydrating solution, and tissue transformation (hydrogel)-based clearing methods 4. The solvent-based methods use RI matching solutions of 1.5 RI, whereas RI of matching solution in the other three methods ranges between 1.43-1.47 on average. The strength of lipid removal also contributes to tissue transparency resulting from diverse clearing methods. In general, solvent, tissue transformation-based methods, and most hyperhydrating solution-based methods require substantial lipid removal for excellent transparency, while in simple immersion-based methods, samples are immersed in gradient-concentration aqueous solutions with a high RI, thereby replacing water and matching the RI of tissues with the lipid retention. Over the years, the basic principles and formulas of solvent-based clearing methods have not changed significantly. The methods make the RI of tissues homogenous through dehydration, delipidation, and RI matching with organic solvents. Compared with initial organic solvent removal methods, such as BABB and DBE 9-11, a series of modern techniques derived from 3DISCO can provide practical solutions for whole-mount immunolabeling, fluorescence preservation, and whole-body imaging 6, 12-16. ECi (ethyl-cinnamate)-based RI matching strategy provides a nontoxic solvent-based clearing method that can clear tissues with calcified bones and retain fluorescence label over weeks 17. Two other clearing methods based on high RI aqueous solutions and hyperhydrating solutions, especially the former, are generally limited to mouse embryos, newborn mouse brains, or thin tissues (thin or low lipid-content samples). The method based on high RI aqueous solutions has high lipid and protein compatibility and a significant ability to retain micro-morphology 3, 18-20. In recent years, a hyperhydrating method CUBIC and its modified techniques have been reported, enabling faster optical clearance of large specimens using hyperhydrating solutions (including the entire mouse body and neonatal marmoset hemispheres) 8, 21-24. The hyperhydrating protocols for the whole mouse body or larger samples usually rely on cardiac perfusion 8, 21, 22, 24, similar to the high RI aqueous solution-based method FRUIT 25.

Unlike other clearing methods, in tissue transformation-based clearing schemes, substances such as acrylamide are introduced to form tissue-hydrogel hybrids to preserve the anatomical structure, proteins and nucleic acids during the process of tissue clearing. The pioneering technology in this field is CLARITY, which is a typical active clearing method that can rapidly extract lipids by electrophoresis 26. However, it is challenging to properly control the electrophoresis current to avoid the degradation of tissue-gel hybrids and the loss of epitopes 27. Thus, effective passive hydrogel-based clearing methods optimized by environmental conditions (temperatures, pH, etc.) have been introduced 28, 29, in addition to active processes mediated by perfusion, modified electrophoresis conditions, or continuous convective flow 29-31. In addition to the general tissue transformation-based clearing method, an extended clearing technique termed Expansion Microscopy (ExM) is proposed, which expands the mixed specimen in a hydrogel-mediated isotropic manner so that conventional optical microscope can be used to obtain images with nanoscale resolution 32-34. Tissue transformation-based methods, CLARITY and ExM can combine with RNA FISH (fluorescence in situ hybridization) or RNA in situ sequencing techniques, which provides an effective strategy for multiplex imaging of the transcriptome in large tissue that preserves the anatomical structure 35. Additionally, due to the native antigen preservation ability derived by tissue-hydrogel hybridization, the hydrogel-based clearing methods are suitable for multi-round immunolabeling, enabling protein composition imaging 36. At present, modern clearing technology still focuses more on the transparency of large tissues, RNA imaging in large-volume samples, and scalable immunolabeling of intact tissues, thus advancing in-depth research on many biological issues from multiple perspectives.

The transparency effect of optical clearing methods is often demonstrated in mouse embryos and brains as they are essential in neuroscience research. Due to the difference in clearance principle and RI matching, various clearing methods are different in many aspects. For example, the simple immersion-based technology is the mildest. It can clear the mouse embryos and brains of newborn mice without defatting and deforming, so it is often used to observe the morphological details of neurons 3, 19, 20. Other hydrophilic methods, hyperhydrating methods not only retain the excellent biocompatibility of high RI aqueous solutions but also have the ability to clear large tissues. However, the incubation time is longer. Generally, using the simple immersion and hyperhydrating solution-based methods can quickly clear smaller tissues very well. In addition, CUBIC, solvent-based method, and tissue transformation-based method can make large samples optically transparent. CUBIC methods induce tissue expansion during the procedure, and CUBIC-X that induce noticeable volume expansion may eventually mediate the scanning of the intact brain with subcellular resolution 37. Tissue transformation-based methods can also lead to tissue swelling, which in some cases may be enhanced to improve the resolution of ordinary microscopes. On the contrary, solvent-based clearing methods introduce severe shrinkage of the tissue, and this character can be used to reduce the imaging time of large samples. To select a suitable clearing method, the volume change of cells or tissues, the retention capacity of various biomolecules in tissues, time consumption, and total cost may be critical considerations. Notably, some clearing methods mentioned above can be applied to thick samples from the human brain. There are also some specialized methods invented for clearing adult human brain samples, such as MASH for cytoarchitectonic labeling and clearing of human cortex samples 38, SHANEL that enables whole human organ clearing (including the intact adult brain) 39, as well as hFRUIT updated from original FRUIT clearing protocol enabling lipid-preserved clearing and DiI labeling of thick human samples with high myelin content 40. These optical clearing methods have been applied successfully, and they can promote the understanding of the brain from various perspectives. Next, we will discuss the studies based on the optically transparent brain tissue in detail.

## Neural circuit

**Figure 1:**
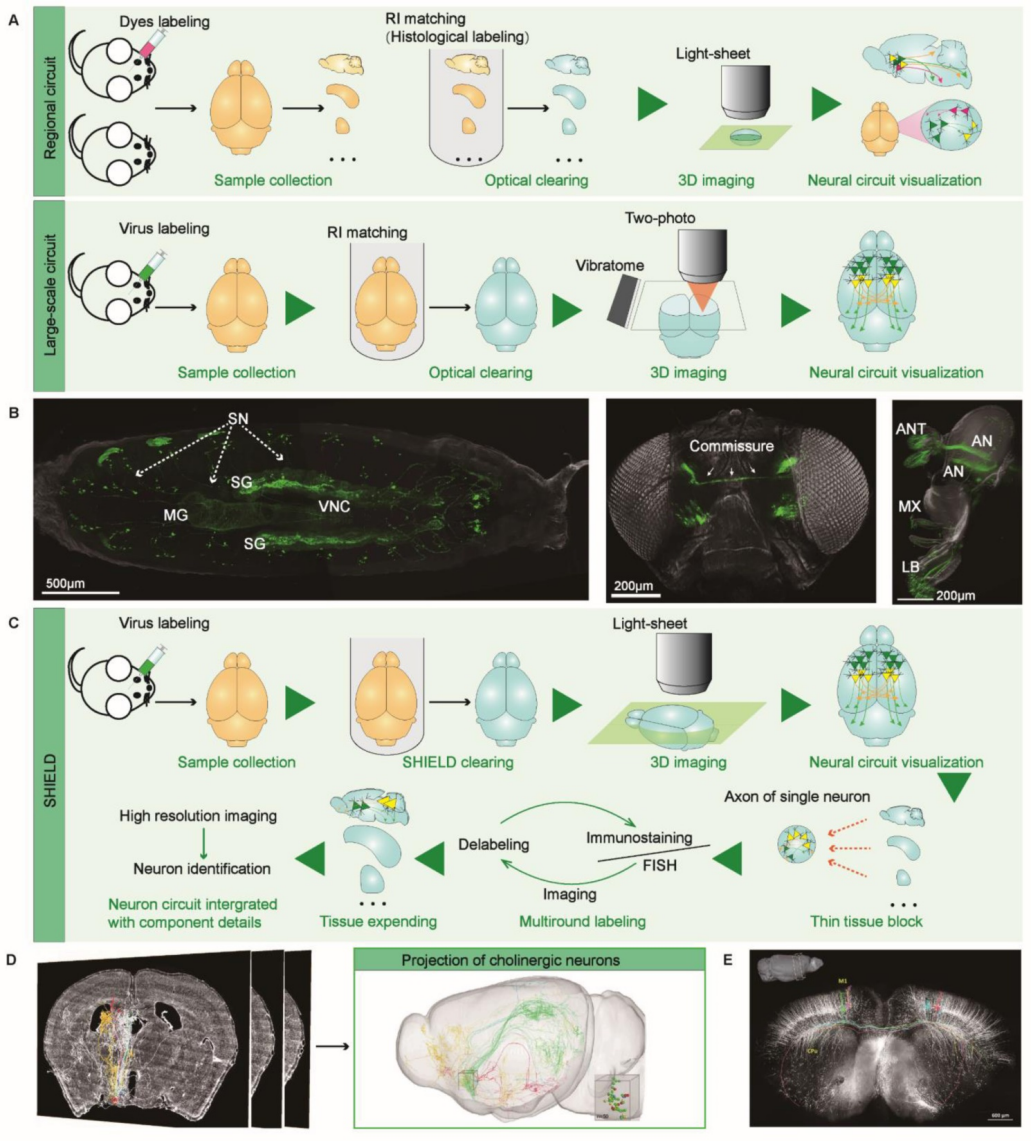
Neural circuit analysis with optical clearing or MOST. (A) Schematic illustration of using optical clearing technology to visualize the regional or long-scale neuron circuits. (B) Visualizing long-range connections of peripheral sensory and central neurons in the FlyClear-based optically cleared Drosophila melanogaster. The connection details in prepupa, visual system, and chemosensory systems are acquired with aspheric ultramicroscope from GFP-expressing flies. Adapted from 41, copyright 2018 nature publishing group. (C) SHIELD enabled the three-dimensional rendering of the brain-wide neuron circuits by integrating the information obtained from local FISH, IHC 45. (D) The three-dimensional reconstuction of brain-wide projections of cholinergic neurons in MS/VDB labeled with AAV-CAGFLEX-GFP virus. Adapted from 47 with permission, copyright 2018 National Academy of Sciences. (E) fMOST-based reconstruction of the coronal plane imaging of GFP-M line mouse brain. The neurons with long-range projection were indicated in different colors. Adapted from 49, copyright 2015 Frontiers.

The neural circuit composed of neurons connected by a synapse is the anatomical structure and functional network of the brain. They reflect the pattern of neuron connection and the transmission of activity between neurons. Visualizing the neural circuits of the entire intact tissues helps provide an anatomical basis for signal coding and routing research, and tissue optical clearing with submicron resolution seems to be an ideal method. However, due to the contradiction between tissue transparency, fluorescence retention, and tissue morphology invariance, the neural circuits are visualized by the optical clearing method to light up specific paths for particular tasks (Figure 1A, top panel). Ke et al. constructed a near-complete wiring diagram of mitral cells associated with a single glomerulus in the mouse olfactory bulb via the optical clearing method, facilitating the study of non-redundant odor encoded by these neurons 3. The visualization of neural circuits in other brain regions (such as the mouse visual system) through optical clearing techniques has also been reported 20. Recently, a novel protocol called FlyClear was proposed, which combines tissue clearing, ultramicroscopy, and data analysis to visualize the remote connections of peripheral sensory and central neurons in whole transparent Drosophila flies (Figure 1B) 41.

As for mapping large-scale neural circuits in the intact mouse brain (Figure 1A, bottom panel), Economo et al. proposed a platform for whole-brain imaging that combines serial sectioning of tissue vibrators with sorbitol-based optical clearing and sparse viral labeling techniques 42. To demonstrate the effectiveness of this approach, single axon fibers and thin axon collateral branches of multiple neurons from the motor cortex of intact mouse brains were reconstructed to draw long-distance projections of single neurons 42. Later, this team improved the above method and developed a semi-automated, high-throughput reconstruction pipeline. Through this optimized approach, more than 1000 projection neurons from different mouse brain regions were reconstructed, and the discovery of new tissue principles that control remote connections can be facilitated 43. Lin et al. have also introduced a similar approach integrated with a dual-adeno-associated virus expression system, which can reconstruct neuron circuits with cell type and projection specificity 44. Identification of the molecular phenotypes of cells with similar axonal projection patterns and their downstream target cells is essential for understanding neural circuit functions and information transmission patterns. Unfortunately, integrating specific molecular phenotype information of cells with the overall view of diverse brain regions remains challenging. SHIELD, an optical clearing method, allowed a rapid, multiscale comprehensive analysis of neural circuits in the mouse brain hemispheres, including molecular phenotypic neurons from local tissues. Performing viral labeling, SHIELD, FISH, followed by immunostaining and tissue expanding, the authors comprehensively characterized the neural circuits that start from the outside of the globus pallidus external (GPe) and project to multiple brain regions (Figure 1C) 45. Luo et al. have developed a micro-optical sectioning tomography (MOST) system to overcome the mesoscale limitation of brain-wide neural circuits imaging, providing a three-dimensional dataset of the whole mouse brain with a one-micro voxel resolution to distinguish structural details of neurons 46. Compared with micro-optical sectioning tomography (MOST) (Figure 1D-E) 47-49, there is still a long distance to go to achieve precise visualization of neural circuits based on optical clearing. Besides the clearing technology itself, the bottlenecks of light-sheet microscopes, including low resolution and high spherical aberration interference, limit the visualization of thin nerve fibers. Thus, multi-directional selective planar illumination microscopy (mSPIM) has been explicitly developed and become the primary imaging equipment types 50.

## Brain atlas

**Figure 2:**
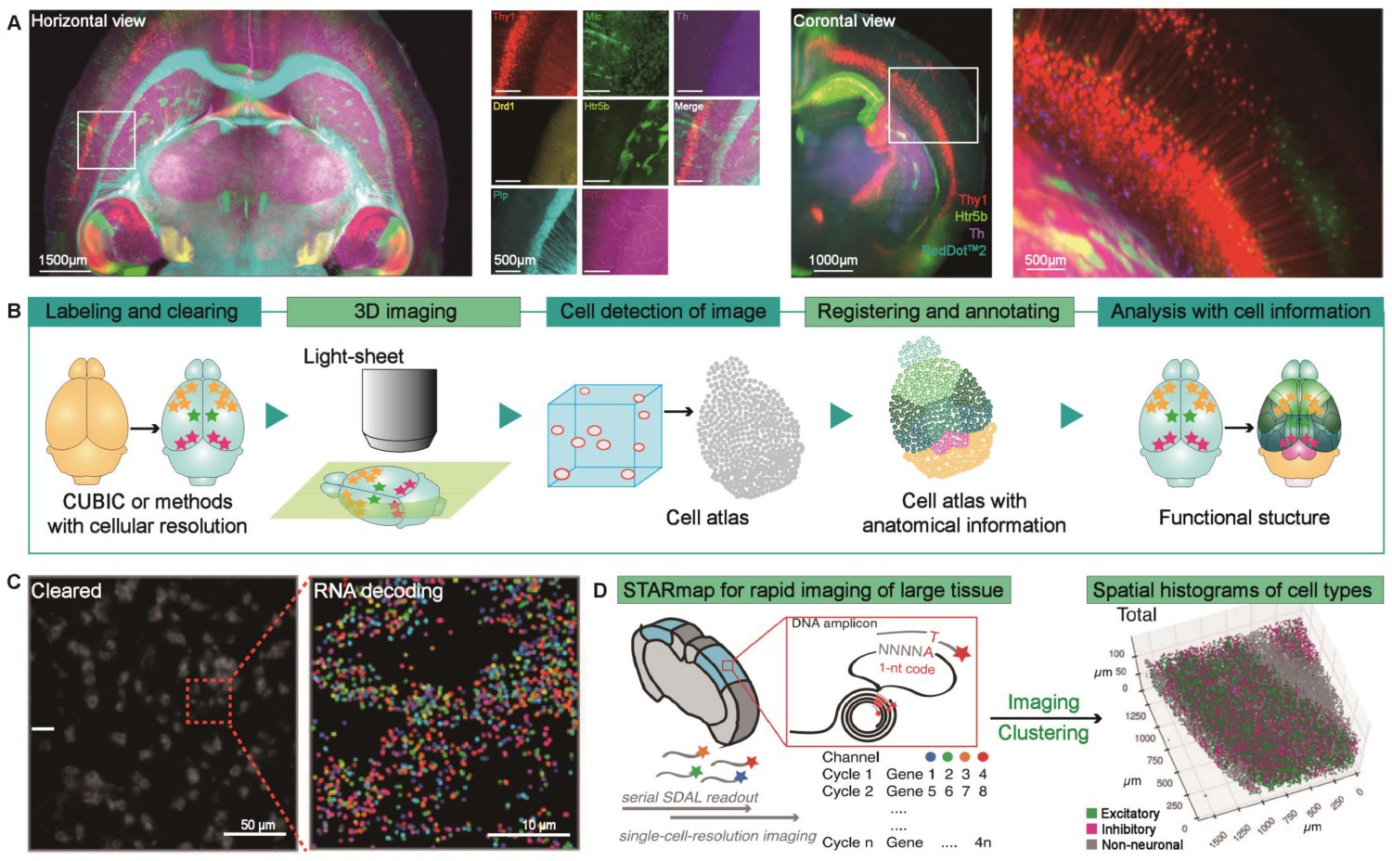
Brain atlas analysis by transgenic model or histological labeling in cleared tissues. (A) Multiplex imaging of mouse brains expressing seven different genetically modified fluorescent proteins. Adapted from 22, copyright 2018 Elsevier Inc. (B) The pipeline of the whole-brain profiling based on the brain cell atlas. CUBIC or other clearing methods with single-cell resolution provide a novel brain atlas, permitting brain types or activities analysis at the single-cell and comparing brains in various physiological conditions after registering to the reference atlas 23. (C) MERFISH measurement of a small portion of a 10-μm-thick mouse hypothalamus slice using a probe set that encodes specific RNA barcodes for 130 RNAs. Single optical section image of the cleared mouse hypothalamus slice (left); Zoom-in of the region indicated with the red dashed box. Adapted with permission from 55, copyright 2016 National Academy of Sciences. (D) Overview of STARmap for rapid volumetric sequencing of large tissue and its application to visualize the cell types in mouse visual cortex volumes. Adapted with permission from 56, copyright 2018 The American Association for the Advancement of Science.

Brain atlas is the key to analyzing whole-brain microscopy images and sharing data in various other experiments 51. It can be an effective anatomical platform that combines neural projection analysis, protein expression analysis, and transcriptome data to map the neural circuits, cell populations, and neural activities of the mouse brain, thereby allowing comprehensive evaluation of the whole brain 23. Susaki et al. reported some research results based on CUBIC protocol combined with mouse brain atlas. The basic scheme of CUBIC informatics they used can be summarized as follows: register the whole-brain nucleus-stained image to the reference brain atlas to create a registration matrix. Then, the resulting matrix is applied to the signal images from the same cleared samples 22-24. Initially, they visualized and quantified the environmentally induced neural activities in Arc-dVenus transgenic mice 24. In their follow-up research, the CUBIC protocol was optimized to achieve fluorescent protein (FP) compatibility so that the distribution of neural subtypes in the entire mouse brain could be visualized through genetically encoded FPs (Figure 2A) 22. Recently, the team developed an editable atlas based on mouse brain cells to easily integrate different cellular information such as cell types (Figure 2B) 23, providing a sharing platform for an infinite amount of research data.

However, the studies we discussed above are all based on transgenic modified mouse models, limiting the scope of the brain atlas. Recently, high-resolution imaging of molecular markers in intact tissues through protein and nucleic acid markers has attracted widespread attention. It may provide better scientific opportunities for brain atlases and enable the analysis of protein expression profiles and transcripts in the brain, thereby enriching the study of cell activity and phenotype distribution in the mouse brain. A critical point in determining the analytical power of this approach is the ability to re-label for multiplex imaging. SWITCH can simultaneously perform protein labeling reactions in intact tissues and produce heat and chemical-resistant samples. Murray et al. used SWITCH to accurately co-register and label 22 individual masses and use unbiased combinatorial protein expression profiles of specific proteins in six cell types to deconstruct the human visual cortex, thereby providing high-dimensional information for understanding the nervous system at multiple levels 28. In addition, understanding how to process neural information will benefit from the visualization and quantification of neuronal activity throughout the intact brain 52. However, it is still a challenge to expand the clearing methods for whole-brain immunolabeling within reasonable processing times. Renier et al. developed a size-preserved method termed iDISCO+ for overall labeling and automatic map registration and a platform termed ClearMap for automated analysis of the neuronal activity in the intact brains 52. This pipeline can visualize the expression of immediate early genes (IEGs) in the whole brain to reflect the latest activity of neurons. Here, they combined axon tracking with IEGs localization, revealing the entire brain functional connection pathways of mice with nurturing behavior 52.

So far, the tissue optical clearing methods developed for the analysis of molecular characteristics in the brain mainly focus on the target proteins for immunolabeling using transgenic models or antibodies. We know that volumetric nucleic acid labels can work relatively flexibly to obtain large amount of information about neurons 35, 53. Due to the rapid hydrolysis of RNA during dilution, most clearing techniques are incompatible with RNA staining. However, the limited specificity and availability of antibodies and the vast potential of volumetric nucleic acid markers in querying molecular information have attracted the attention of researchers to this approach.

The classical approach for imaging RNA in intact tissues is to perform whole-mount fluorescence in situ hybridization (FISH) in cleared samples. Yang et al. showed that PACT is compatible with single molecular FISH (smFISH), and found abundant expression of β-actin transcripts and mRNA in 100 μm-thick PACT-cleared mouse brain sections 29. Later, Sylwestrak research group expanded the sample size to a few millimeters thick mouse brain block through the carbodiimide-optimized CLARITY clearance protocol so that multiplexed transcriptional analysis can be performed in thick brain tissues at a cellular resolution 35. A significant milestone will be detecting multiple distribution patterns of several microRNAs with known functions in the mouse brain. These small non-coding RNAs are essential for the regulation of post-transcriptional gene expression 35. Therefore, this method may discover comprehensive spatiotemporal modulation mechanisms from molecules to cells to the entire brain in the future. Recently, the functional integration of iDISCO+ and in situ hybridization has been evaluated. Researchers have used this volumetric FISH protocol to assess the effect of GR overexpression on the basal levels of Sst mRNA in the mouse cortex 54. To extend smFISH to high-throughput transcriptome profiling and system-level imaging, multiplexed smFISH based on combined oligonucleotide probes encoding RNA-specific barcodes has been developed. For example, Moffitt et al. reported an optimized MERFISH imaging platform, which can analyze 140 kinds of RNA using 16-bit codes, four rounds of hybridization, and four-color imaging per round (Figure 2C) 55. Another typical method is three-dimensional in situ sequencing, which requires shorter RNA probes and is more flexible than FISH-based methods. Wang et al. used this approach to locate the 3D spatial cell typing of mouse V1 clusters from 28 genomes by STARmap, a volume sequencing method combining hydrogel histochemistry and optical clearing (Figure 2D) 56. In short, the brain atlas generated by the optical clearing methods complements cellular and even molecular perspectives, and various labeling methods can reveal different forms of biological information. With the development of these techniques, more information about the structure and function of mammalian brains will be discovered.

## Visualizing morphological details of blood vessels in the brain

Combined with microscopic imaging technology, the tissue optical clearing method enables us to visualize various morphological details in the brain, thereby studying the relationship between morphological structure and function, and revealing the pathological changes surrounding the occurrence of diseases. The cerebrovascular system provides rich nutrition for brain tissues and removes metabolic waste from the brain. Thus, it is crucial to understand the structure and molecular details of the cerebrovascular system. Blood vessels are mainly composed of endothelial cells, smooth muscle cells (arteriovenous blood vessels), and pericytes (capillaries). Blood vessels can be labeled with various cell surface markers, such as α-smooth muscle actin (α-SMA). α-SMA is expressed during vascular remodeling and can mark arteries and arterioles as well as neuron-glial antigen 2 (NG2), which is a sign of pericyte activation in pathological conditions. In addition, exogenous markers such as lectins are also useful in labeling blood vessels. When used in combination with optical clearing techniques, these markers mentioned above usually require cardiac perfusion to mark and visualize the complete vascular network. Hama et al. used cardiac perfusion to label the entire blood vessel and use Scale to eliminate transgenic mice's hippocampus 57. The vascular niche for neural stem cells (NSCs) in the subgranular zone (SGZ) of the hippocampus dentate gyrus has been studied, which provides the potential to obtain a comprehensive 3D view of the interaction between NSC or other biological niche and cerebral blood vessels in this region 57. Recently, a molecular phenotype compatible method SeeNet was introduced to visualize the cerebrovascular networks accurately. The method combines a thermally controlled vessel casting method with a bile salt-based tissue clearing technique to optimize the function of observing cranial blood vessels. Compared with the classic vascular casting or lectin transcardial perfusion method, SeeNet shows close to 100% vascular coverage, thus becoming a promising tool to reveal unknown vascular access and complete the cerebrovascular network's connectivity 58. The “skull optical clearance window” presents a novel strategy to image cerebral blood vessels of mice in vivo. Zhao et al. developed a skull optical clearing window for cortical imaging with synaptic resolution 59. It is possible to visualize cortical blood vessels and nerve cells during specific activities or diseases due to its safety in experimental mice. Later, this group proposed a novel method for large skull optical clearing using clearing agents termed USOCA, which can achieve optically transparent regional or bi-hemispheric skull in 15 minutes 60. However, it is still a challenge to excavate valuable information from the three-dimensional clearing imaging datasets. Todorov et al. developed a machine learning-based method called VesSAP to segment brain blood vessels and quantify the characteristics of the whole-brain blood vessels after the samples are registered in the Allen Mouse Brain Atlas 61. It is also expected to develop more automatic analysis pipelines to accelerate the comprehensive research of cerebrovascular networks. Additionally, non-invasive optical in vivo imaging methods, such as optical coherence tomography and laser speckle imaging, enable the evaluation of cerebral blood flow in the cortex of rodents, reflecting the disturbance of physiological activities in the organisms 62, 63. Therefore, combining these non-invasive techniques with optical clearing techniques can provide abundant molecular information that will enable a more comprehensive understanding of many disease mechanisms. Wei et al. have done attractive work on the application of this combination. Taking advantage of the skull/skin optical clearing window, they monitored the blood flow and oxygen change of cerebral and cutaneous vessels in diabetic mice by combining laser speckle contrast imaging and cutaneous hyperspectral imaging and suggested that the abnormality of cutaneous blood oxygen might be an early indication of cerebrovascular dysfunction caused by diabetes 64.

In the future, more innovative research based on the combination of optical clearing methods and in vivo imaging techniques are expected, and the depth of in vivo imaging is expected to increase further. As for human samples, Harrison et al. reported a novel method based on 3DISCO clearing and fluorescent Lycopersicon esculentum agglutinin labeling to visualize the cerebral cortical vasculature from the long-fixed human brain 65. This method permits the visualization of the parallel arrangement of the vasculature in the cortex and further assesses the physiological or pathological brain states 65. Additionally, optical clearance of the human dura mater is a potential method to aid the post-mortem investigation of human head injury since dissection of dura mater after skull removal may introduce other damage of brain samples 66. Gong et al. used a modified Nissl staining strategy and MOST system to develop a 3D brainwide cellular and vascular (3D BrainCV) visualization and quantitative protocol to snapshot the detailed picture of the whole brain architecture 67, 68. Recently, MOST was applied vascular analysis in Alzheimer's disease and found the vascular morphological mutation in the hippocampus in the transgenic (APP/PS1) mouse model of AD 69. Although optical clearing methods have shortcomings in imaging resolution compared with MOST, the imaging equipment required for optical clearing technology is relatively common, making it suitable for a variety of laboratory conditions.

## The disease model of brains

### Alzheimer's disease and other cognitive diseases

**Figure 3:**
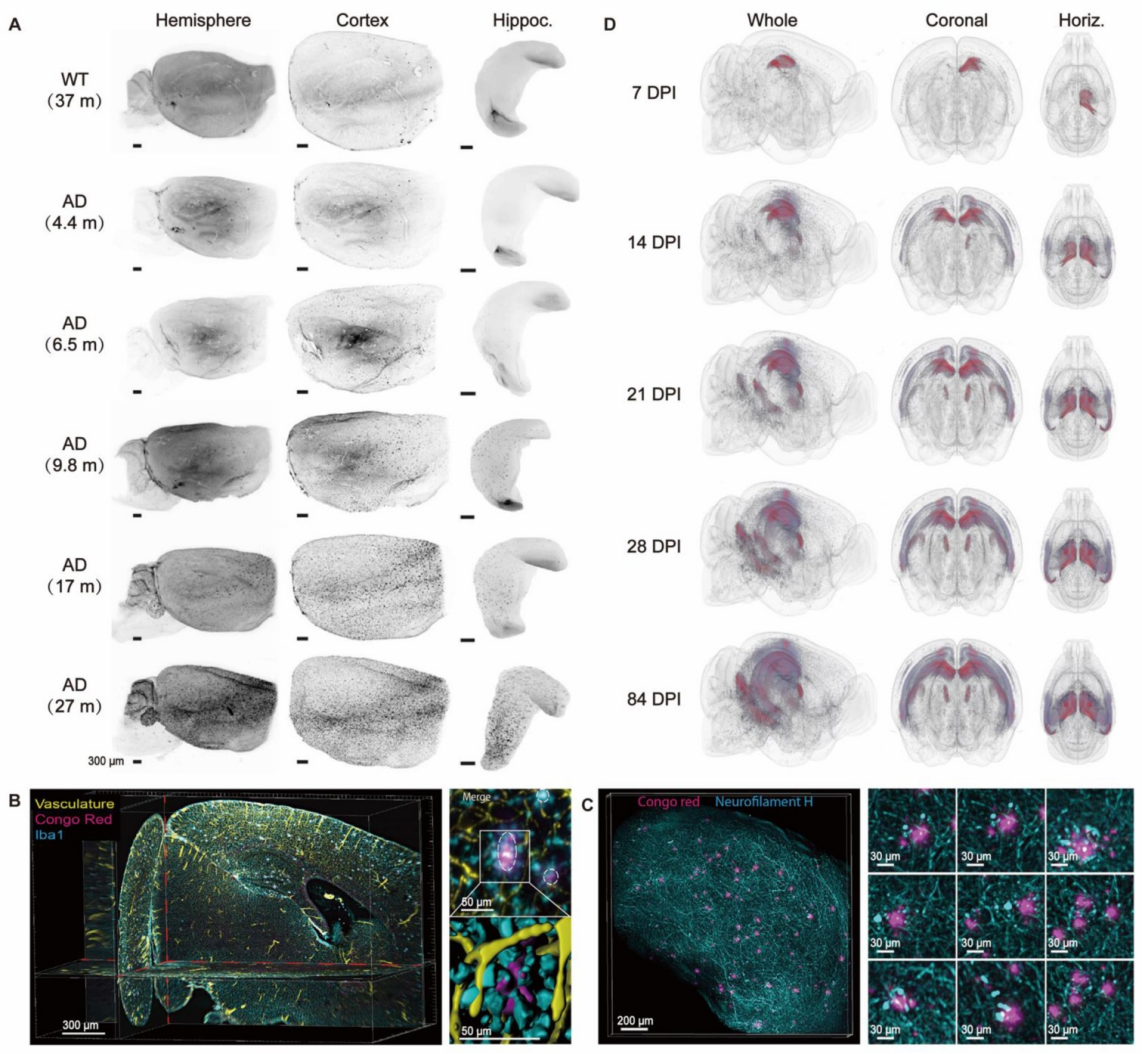
Multiscale visualization of AD pathology across brains. (A) Visualization of the gradual progression of Aβ deposition by anti-β-amyloid plaque staining in cleared old WT mouse brains and 2xTg AD mouse brains at different ages. Adapted from 73, copyright 2016 Elsevier Inc. (B, C) Multiple labeling and three-dimensional imaging of Aβ plaques and relevant cellular or macroscale markers in cleared 2xTg AD brains. Maximum intensity projection of an optical section (1 mm thick) from an 11-month-old 2xTg AD mouse brain labeled for vasculature, Aβ, and microglia (left); Maximum intensity projection of a 10-month-old 2xTg AD cleared mouse cortex (500 mm thick) stained with Aβ and neurofilament H for axonal dystrophy investigation in AD (right). Adapted from 73, copyright 2016 Elsevier Inc. (D) Heatmap plots used for profiling the increase of hyperphosphorylated tau pathology in cleared K18-injected Tau.P301L mouse brains over time. DPI, days post-injection. Adapted from 78, copyright 2018 Frontiers.

Alzheimer's disease (AD), one of the most common causes of dementia, is a progressive, neurodegenerative cognitive disease. The typical pathological characteristics of AD are macroscopic cortical atrophy and brain weight loss as well as neuropathy, such as extracellular amyloid β (Aβ) peptide deposits, neurofibrillary tangles (NFTs) mediated by intracellular tau aggregates, and reactive glial hyperplasia under the microscope 70, 71. A precise diagnosis of AD requires a complete medical examination, including neurological and blood examinations and brain imaging. Routine structural neuroimaging techniques such as CT and MRI reveal the anatomical changes of brains in the final stage of AD. Functional neuroimaging, including functional MRI (fMRI) and PET, provides abnormalities in brain metabolism in prodromal or even presymptomatic states. In particular, molecular PET imaging reveals how Aβ plaques and tau proteins are distributed, thereby explaining the clinical manifestation of AD 72. However, to understand the detailed pathological features and molecular mechanisms, it is necessary to introduce histological research. Optical clearing involving molecular markers can bridge the gap between the macroscopic view and the microscopic view, thereby providing a three-dimensional histological picture of AD pathology. Recent reports suggest that neurodegeneration and cognitive impairment are mainly caused by amyloidosis. Therefore, it is necessary to understand the spatiotemporal progression of Aβ plaque accumulated in all brain regions and determine the brain regions with early Aβ susceptibility. Liebmann et al. combined iDISCO optical clearing with ClearMap analysis to evaluate the development of hemispheric Aβ plaques in 2xTg AD mice from 4.4 to 27 months. It was found that Aβ plaques firstly appeared in the cortex, and the number and distance between plaques in different brain regions were subsequently analyzed in 3D space (Figure 3A) 73. Another research group used two-photon tomography to image the spatial patterns of Aβ deposition in three AD mouse models, which confirmed the first appearance of Aβ plaques in the mouse cortex 74. Furthermore, Canter et al. used the SWITCH protocol to clear and label intact mouse brains and created a spatiotemporal and network-specific map of Aβ progression in the 5XFAD mouse model, revealing that dysfunctional memory circuits are the core AD pathology 75. They discovered the early papillary body dysfunction and Aβ Papez circuit susceptibility in AD, which has not been found before 75. Recently, the comparison of volumetric multispectral photoacoustic tomography (vMSOT) and optical clearing-based brain imaging has shown highly corresponding Aβ plaques 76. In addition, the Aβ plaque-associated microenvironment also deserves attention to better understand Aβ development. Hama et al. used the clearing method AbScale to immunolabel amyloid plaques specifically and demonstrated that the combination of plaques, microglia cells, and related active inflammation molecules mainly occurred in the early stages of Aβ development in both mouse models and patients 77. They also analyzed the spatial associations between microglia and plaques and found the clustering of microglia around diffuse extracellular plaques 77. The contextual evaluation of amyloid plaques and axonal dystrophy, cerebrovascular network, and tau have been imaged by the immunolabelling-friendly clearing method iDISCO (Figure 3B-C) 73. Applying iDISCO to archived brain tissues from AD patients for tridimensional analysis, a remarkable difference in the morphology, density, and size of plaques was observed among individuals 73. Additionally, the spread patterns of tau pathology can also be mapped by optical clearing. Due to apparent pattern differences of tauopathies between mouse and human, Detrez et al. proposed a controlled model by inoculating pathogenic tau fibrils to specific brain regions of tauopathy mouse model (Figure 3D) 78, providing us a novel strategy for volumetric optical clearing imaging of tau pathology in whole mouse brain. However, the validity of the controlled model still needs further study and discussion.

Other cognitive diseases have also been studied by optical clearing technology. Cerebral amyloid angiopathy (CAA) is an age-related neurovascular disease that usually occurs in the elderly. CAA is fatal due to microbleeds and cerebrovascular amyloid deposits 79. Lo et al. combined optical clearing technique with histochemical staining to visualize cerebral microvessels and pathological features. The results showed that spontaneous microhemorrhages were very close to leaky or ruptured hemorrhagic microvessels caused by amyloid 79. In the study of Parkinson's disease based on optical clearance, the passive CLARITY technology was used to visualize the gap between monoaminergic fibres and substantia nigra striatum in three dimensions midbrain tissue of post-mortem human Parkinson's disease patients 80. It is expected that more advanced labeling and clearing technologies will be developed to provide new insights into neurocognitive diseases at the molecular level in three-dimensional brain tissues.

### Brain tumors and other diseases related to the brains

**Figure 4:**
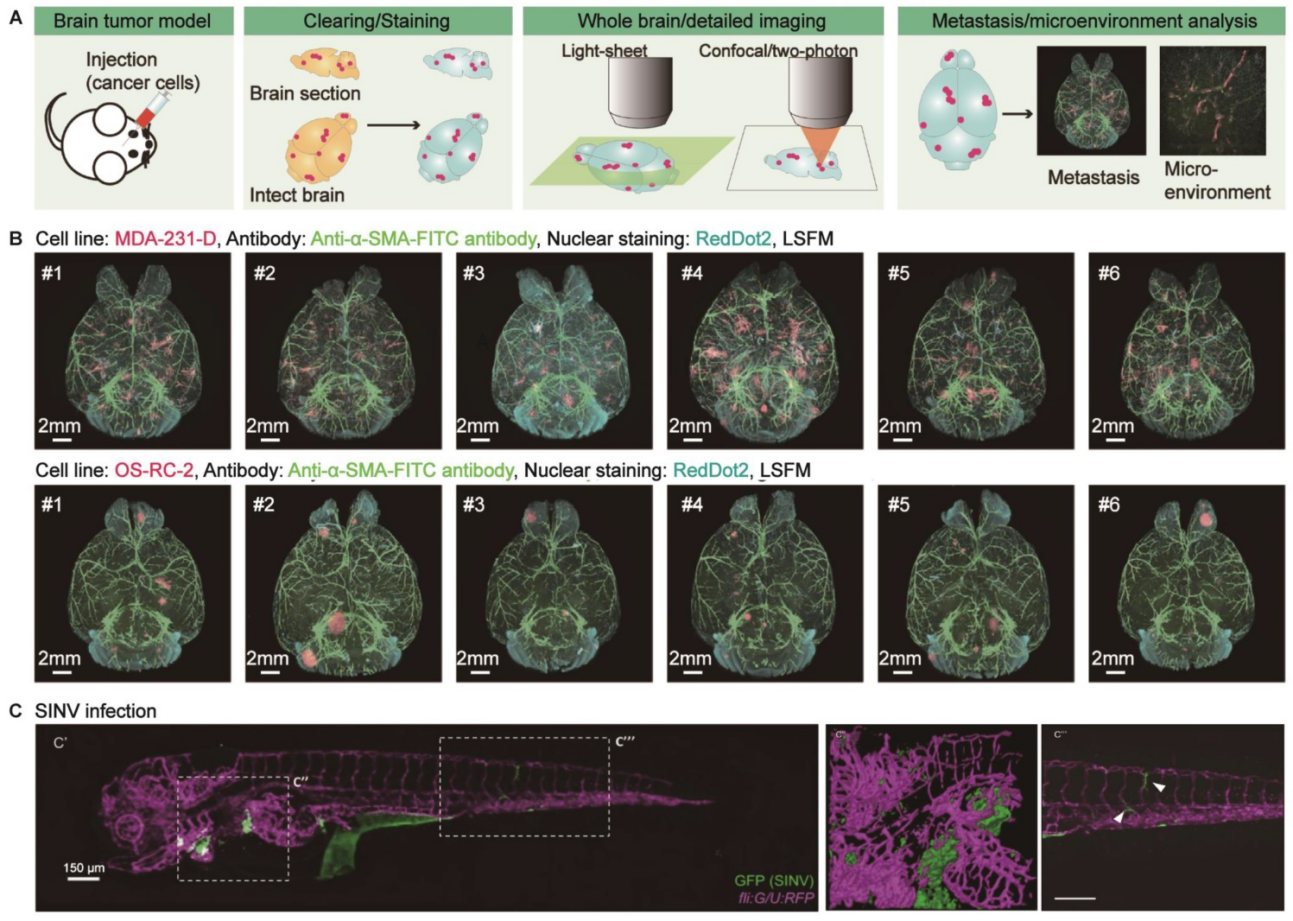
Applications of optical clearing in brain tumors and other intracranial diseases. (A) Schematic illustration of tumor metastasis or microenvironment study in mouse brains using tissue optical clearing technology. Adapted from 83, copyright 2017 Elsevier Inc. (B) Imaging and comparing the metastatic pattern of two experimental brain metastasis models based on CUBIC-cancer analysis. Adapted from 83, copyright 2017 Elsevier Inc. (C) Visualizing the Sindbis virus (SINV) neuroinvasion in CLARITY-cleared zebrafish at 2 DPI (days post-injection). Adapted from 86, copyright 2017 The Company of Biologists Ltd.

Brain tumors are primary tumors originating from intracranial tissues or metastatic tumors that invade the brain from other tissues or organs. Heterogeneity and metastasis are the main factors that lead to poor prognosis. Conventional 2D histological analysis hinders the investigation of tumor microenvironments and metastasis patterns at a three-dimensional level with molecular resolution. However, optical clearing technology provides a promising strategy for thoroughly examining tumor heterogeneity and metastasis (Figure 4A). Glioblastoma (GBM) is the most malignant glioma of various astrocytic tumors and exhibits an aggressive growth pattern. Lagerweij et al. performed in-depth imaging of GBM and cerebral microvessels based on optical clearing technology quantitatively evaluated the infiltration of GBM in the microvascular system and analyzed the pattern of invading along the white matter tract and brain vessels in two glioblastoma mouse models 81. Recently, Yang et al. compared the ability of two-dimensional analysis and tissue optical clearing to dissect the spatial heterogeneity of GBM. The latter has an overwhelming advantage over the former and can be used in cell stemness, microvasculature, and immune cell infiltration in GBM 82. To explore metastatic tumor patterns, CUBIC-cancer analysis on two brain metastasis models statistically clarified the roundness and the formation of vascular-based migration (angiogenesis and co-selective growth) of metastatic colonies (Figure 4B) 83. Furthermore, the heterogeneity of tumors and their microenvironment may involve the underlying causes that determine metastatic growth 84. Guldner et al. combined optical clearing and forest-based random image analysis to perform global metastatic heterogeneity assessment of various tumor morphology, heterogeneous proliferation index, metastasis-related astrogliosis, and vasculature spatial distribution 84. Additionally, tissue optical clearing combined with immunolabeling allows us to visualize the progression of acute brain injury and viral encephalopathy, such as the visualization of immune cells in meningeal lymphatic vessels to improve the pathology of the middle cerebral artery occlusion (MCAO) model 6, the distribution of rabies virus in the brains of infected mice for the study of virus pathogenesis, transmission, tropism, and neural invasion 85, and the neural invasion routes of alphaviruses (Chikungunya virus and Sindbis virus) in zebrafish with CLARITY-based body clearing (Figure 4C) 86. Also, Phillips et al. illustrated that passive CLARITY cleared human cerebellum samples integrated with immunofluorescent labeling of neurons and mitochondrial proteins would promote the understanding of neurodegeneration mechanisms in mitochondrial disease 87. The application of tissue optical clearing in the studies of various brain diseases has not yet hit scale, and the existing researches are mainly focused on the visualization of the disease development and pathological features. Future research should be extended to the molecular level for a better understanding of the causes of diseases in three-dimensional and thus provides opportunities for the development of relevant targeted drugs.

## Development of brain

**Figure 5:**
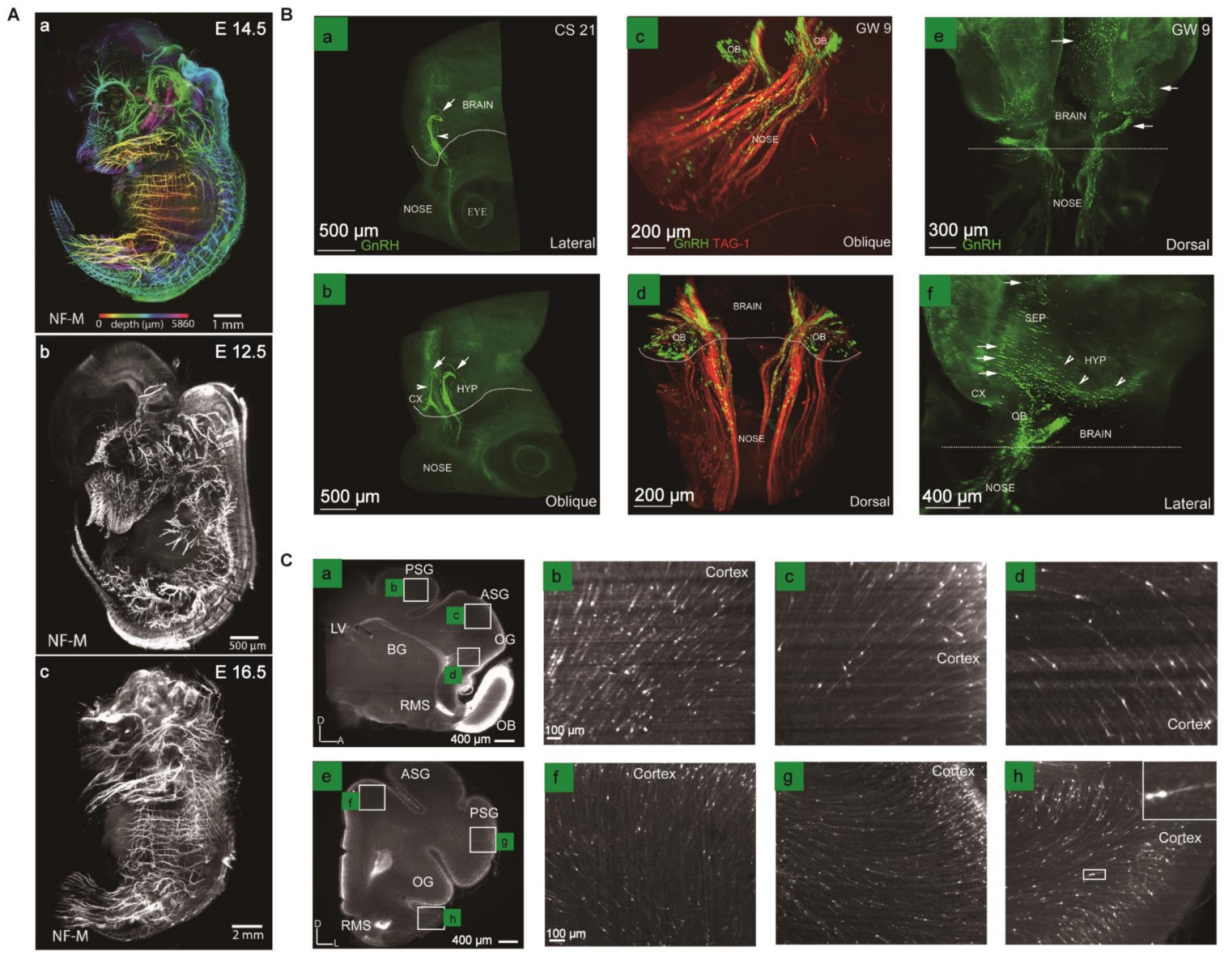
Three-dimensional visualization of the changes in protein expression and the migration of neurons during mouse embryonic development. (A) Intact mouse embryos are labeled for neurofilament (NF-M) at different embryonic development stages. Adapted from 88, copyright 2020 WILEY-VCH Verlag GmbH & Co. KGaA, Weinheim. (B) Profiling the chain-like migration, scattered distribution of GnRH neurons, and the co-localization of GnRH and TAG-1 immunolabeling in CS 21 embryos or GW 9 embryos of human. Adapted from 90, copyright 2016 The Company of Biologists Ltd. (C) Analysis of the transition of SCGN+ cells from white matter streams to the cortex in iDISCO-cleared P20 ferrets. Adapted from 92, copyright 2019 Wiley Periodicals, Inc.

The process of brain development involves many vital stages, such as neuronal proliferation, migration, differentiation, and axon guidance, thereby forming a structured and functional cerebral control center. Tissue optical clearing provides an efficient strategy to make these stages globally visible throughout the developing brains. Some specific proteins, such as neurofilament proteins, play various essential roles in brain development and physiology. Neurofilament proteins form the backbone of neurons endows nerve fibers elasticity. It shows different expression levels in the development of the organism and is associated with the neurodegenerative disease as typical biomarkers of axonal damage. Thus, visualizing the distribution of neurofilament proteins in intact tissues is of great significance. Zhu et.al. proposed an innovative aqueous clearing method termed MACS to reconstruct the three-dimensional nerve distribution in whole mouse embryos at different stages with anti-neurofilament antibody labeling methods, enabling us to evaluate the developmental state of the nervous system (Figure 5A) 88. The PRDM family consisting of 17 orthologous proteins is a potent transcriptional regulator with a wide range of functions on the developmental embryo 89. Woo et al. optimized the passive CLARITY method to clear mouse embryos to investigate the expression patterns of PRDM7 and PRDM12 in intact mouse embryos. The results showed that PRDM7 and PRDM12 highly expressed in some regions in the developing mouse embryo brain, providing evidence for their role in neurodevelopment and congenital diseases 89. In addition to exploring protein distribution patterns, optical clearance technology can also study neuronal migration during embryogenesis. Gasoni et al. used an updated 3DISCO method to provide a quantitative analysis of gonadotropin-releasing hormone (GnRH) neuron origins, differentiation, migration, and their distribution 3D atlas in the fetal brain, revealing that GnRH positive neuron cells have potential roles in non-reproductive function (Figure 5B) 90. Additionally, the migration flow of immature neurons can also be studied in the early postnatal brain of some small animal models, which corresponds to the early development of human brains in this aspect (Figure 5C) 91, 92. Furthermore, other biological stages that occur during neurodevelopment, including axonal guidance, synapse formation, and neuronal death, still need to be further studied through improved clearance techniques, leading to a comprehensive understanding of mammalian brain development.

## Image processing and information extraction tools

Employing optical clearing techniques to observe intact tissue provides a convenient and multi-dimensional spatiotemporal method and brings a significant challenge to researchers: how to process and extract biological information from the large acquired images. However, the size of datasets from cleared brain tissues is usually between a few gigabytes to terabytes. These data are generally labeled with multiple probes conjugated with various fluorescent tags, which places high requirements on hardware and software. Generally, it is necessary to analyze the three-dimensional microscopic image on the workstation with superior calculation performance under procedures including compression, reconstruction, segmentation, and registration of images. Some companies have developed commercially available software, such as Amira, Imaris, Image-Pro Plus (IPP), arivis Vision4D, covering the entire analysis pipeline mentioned above and providing essential general-purpose tools to extract information for related biologists. Some of them are integrated with developer packages; for example, the Amira developer package allows C++ to create new custom modules. However, commercial software is usually expensive and relatively unsuitable for a personalized analysis of the images. Thus, many open-source analytics platforms have been released for 3D image evaluation. Fiji (ImageJ, based on Java) and Cellprofilter (based on Python) are two leading tools that provide more flexible solutions by developing plugins edited by corresponding compilers, such as Simple Neurite Tracer and Sholl Analysis in Fiji for neuron analysis. However, the module for three-dimensional reconstruction and visualization of Fiji is less powerful than other commercial software or dedicated pipelines developed for the reconstruction and analysis of three-dimensional images. Fiji is developed by contributors worldwide and is less modular and specialized, usually used for two-dimensional image analysis.

Furthermore, many researchers have proposed novel algorithms in their particular research for more accurate and high-throughput automatic analysis of optically transparent samples, like RINZO algorithm for measuring the distance from target to the closest nucleus in the intact tissues 57, CUBIC-Atlas associated codes for constructing single cell-based mouse brain atlases 23, automated platform termed ClearMap for mapping neuronal activities 52, as well as DeepMACT for automated quantification of cancer metastasis of various organs in entire cleared mouse bodies at a cellular resolution 93. In general, the former two tools can satisfy the general requirements to extract biological information, such as vascular diameter, neuron branch, and roundness of Aβ from three-dimensional imaging data. However, when the datasets are relatively massive or complicated, it is necessary to develop specific processing programs to enhance image processing efficiency and propose personalized analysis tools to improve the understanding of structures and functions of the investigated tissues. Deep learning may be a promising method, which avoids the traditional algorithm-based pipeline to some extent and achieves high analysis accuracy. In addition, a deep learning-based analysis generally provides superior scalability. For instance, DeepMACT proposed by Pan et al. for quantification of cancer metastasis may apply to other targets, such as Aβ plaques, if the users can provide such data.

## Conclusion and future perspectives

Based on the previous discussion, the clearing methods can be selected according to the experiment purposes, tissues, transparent time, overall cost, etc. Notably, the tissue transformation-based clearing method expands the volume of samples through gel-based swelling so that the microstructure of thick-walled tissues can be visualized under optical microscopes at a conventional resolution, which will bring new ideas for optical clearing technology. For instance, the morphology of RNA and neuronal detail could be visualized with expansion microscopy 94, 95. However, to image microstructures with super-resolution in the entire brain space, there are still some problems that need to be solved, such as the isotropic expansion of tissues and the imaging speed of large-scale samples for optical microscopes. Because of the massive data and relatively low processing rate of the current analysis platforms, these methods are usually limited to small tissue samples. Researchers reported the tissue optical clearing in human brain samples with thickness up to several millimeters 40, 96. Due to the scarcity of human brain samples and the limitations of immunolabeled probes, it is necessary to explore more efficient tissue clearing and labeling strategies to provide valuable references for deciphering human brains.

The combination of optical clearing methods with flexible labeling and optical microscopy techniques provides researchers with an effective strategy to image the brain at the cellular or subcellular resolutions. Previous researchers focused on functional neural circuits in the mouse brain and neuron characteristics and activities displayed on the brain map. Recent studies have reconstructed the remote connection of more than 1000 neurons and map single-cell characteristics to the brain atlas 23, 43. In the future, we hope to track neuron projections of more than 100,000 cells (about 0.1% of all calls in the mouse brain) in the whole brain via improved sparse labeling technology 43. The detailed classification of neurons and the description of the corresponding neuron activities can be realized by combining the brain atlas.

Regarding the maps of brain atlas, multiple optical clearing methods compatible with protein and RNA enable us to achieve comprehensive structural and functional brain mapping, and detailed correlating of transcriptional activities in individual cells and different brain regions. In addition, the use of the optical clearing method to study various disease models is also of great significance. This technique provides a multi-dimensional perspective (macro and micro scales, multiple potential molecular markers related to target diseases, etc.) to reveal the process and pathogenesis of diseases and facilitates the development of drugs that target specific pathogenic molecules.

Currently, tissue clearing technique has been widely used in AD research, but the applications in tumors and other brain diseases are still relatively rare. The immunolabel-compatible blood vessel morphology and changes in cell morphology and spatial distribution complement the pathological studies based on macroscopic imaging and 2D tissue sections, thereby providing new insights to reveal disease progression in principle. The traditional tissue optical clearing methods are only suitable for in vitro tissue imaging. The safe and stable skull optical transparent window technique permits in-depth brain imaging in vivo. However, due to the profound limitations of this method, more studies have been conducted on the isolated brain samples from various individuals at different stages of diseases, thereby providing an effective research strategy to study brain neurodevelopment and disease progression.

**Figure 6:**
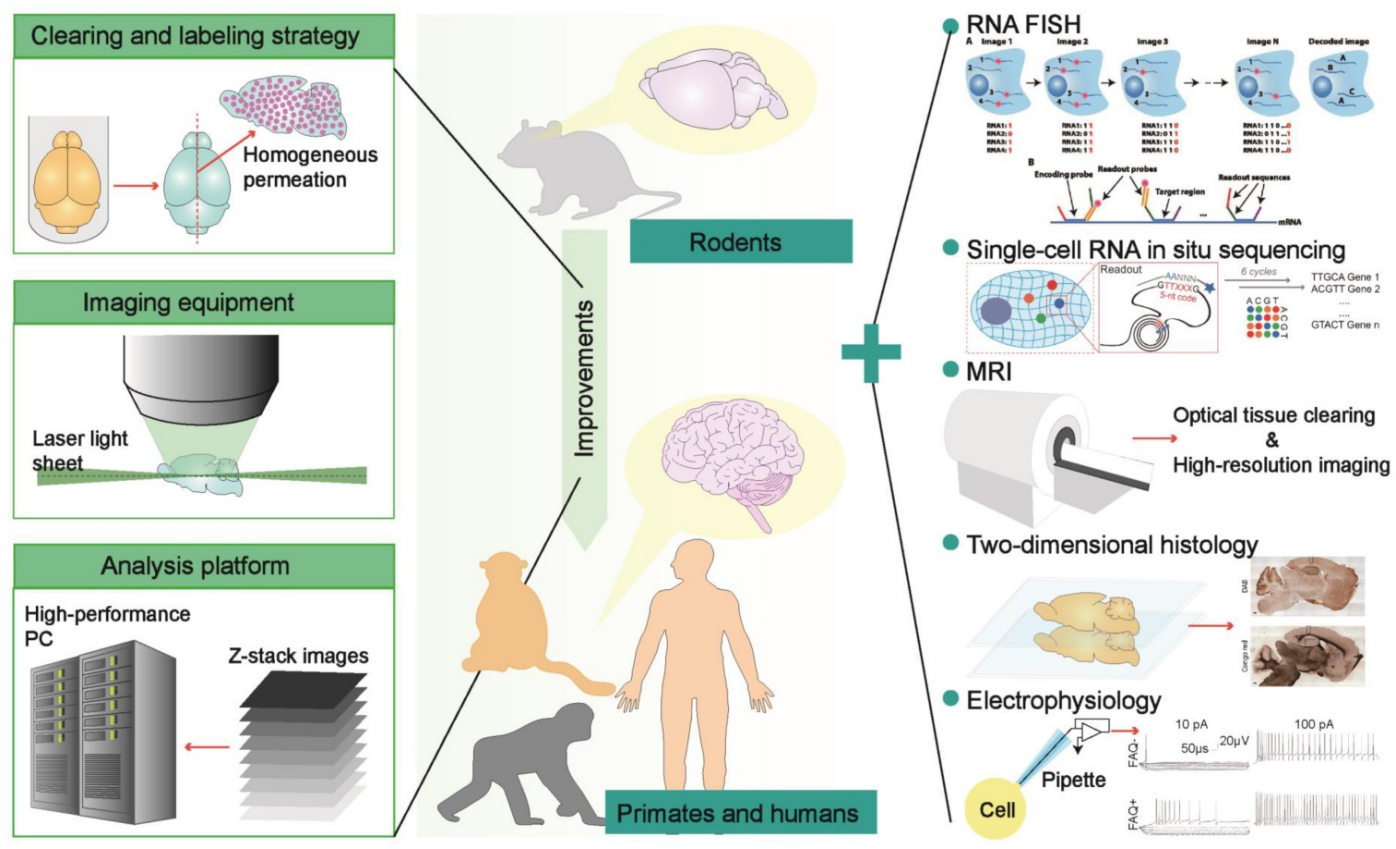
The potential optimization strategies of optical clearing methods for primate brain transparency and some meritorious technologies that can be combined with optical clearance for detail biological profiling. Besides clearing and labeling strategies developed to meet the large size of experimental specimens, light-sheet microscopes enabling large sample imaging and analysis platforms enabling mass dataset processing are also necessary for the optical clearance brains from primates (left). Furthermore, some histological techniques like FISH and other classical methods such as MRI and patch-clamp are expected to integrate with tissue optical clearing technology to provide a comprehensive perspective. Adapted from 55, copyright 2016 National Academies of Sciences, 56, copyright 2018 The American Association for the Advancement of Science, 73, copyright 2016 Elsevier Inc., 75, copyright 2019 nature publishing group.

In summary, the innovations of optical clearing technology are still expected at present. Many researchers are committed to developing tissue optical clearing methods suitable for primate brains or thick brain sections, hoping to achieve useful whole-brain profiling at the cellular and molecular level 39. The coordinated improvements of rapid and homogeneous labeling methods for various biomolecules, light-sheet imaging equipment suitable for large-scale tissues, and analysis platforms of massive imaging data are essential for global analysis of primate brains (Figure 6). Because the labeling and clearing technologies for large samples are difficult to achieve in a short period, and the human specimen is rarely available, we need flexible rodents and primates genetic or surgical models to accurately recapitulate the pathological characteristics of various human diseases 73, which may expand the applications of tissue optical clearing technology. The usage of multiple-round immunolabeling and imaging to develop tissue optical clearing technology inspires the combination of optical clearance with FISH or single-cell RNA in situ sequencing to visualize gene expression profiles in three-dimensional space, thereby promoting the molecular definition of cell types and cell activities in brains. The combination of MRI and tissue optical clearing technology is also feasible, such as the combination of in vitro diffusion tensor imaging (DTI) with two-photon laser microscopy to image intact cleared mouse brains for the evaluation of the correlation between the myelin basic protein (MBP) and the fractional anisotropy of white matter 97, as well as in vivo histology using MRI (hMRI) in humans that promises in vivo mapping of cyto- and myelo-architectonics with histological reference data from cleared human brain tissues 96. Although three-dimensional histology mediated by tissue optical clearing has significant advantages over classical two-dimensional histology, the classical histology is still irreplaceable. Many pipelines that integrate spatial optical clearing imaging and two-dimensional profiling are proposed to provide reliable evidence for biological studies from the whole to the local (Figure 6). Furthermore, the introduction of electrophysiological experiments that reveal the excitability of neurons and behavioral experiments that induce various neuronal activities can provide richer information for brain atlas construction. Therefore, it is possible to build a digital brain based on the framework of a three-dimensional brain atlas, which integrates anatomy, neuron morphology, neuron connectivity, cell phenotype, and neural activity information, thereby deepening the understanding of brain function and dysfunction, providing a comprehensive evaluation system for the staging of systemic brain diseases, and assisting the development of targeted molecular drugs.

## Abbreviations

MRI: magnetic resonance imaging
CT: computed tomography
PET: positron emission tomography
RI: refractive index
pH: pondus hydrogenii
ExM: expansion microscopy
FISH: fluorescence in situ hybridization
GPe: globus pallidus externa
MOST: micro-optical sectioning tomography
mSPIM: multidirectional selective plane illumination microscopy
FP: fluorescent protein
IEGs: immediate early genes
smFISH: single molecular fluorescence in situ hybridization
GR: glucocorticoid receptor
Sst: somatostatin
MERFISH: multiplexed error-robust fluorescence in situ hybridization
α-SMA: α-smooth muscle actin
NG2: neuron-glial antigen 2
NSCs: neural stem cells
SGZ: subgranular zone
AD): Alzheimer's disease
Aβ: amyloid β
NFTs: neurofibrillary tangles
fMRI: functional magnetic resonance imaging
vMSOT: volumetric multispectral photoacoustic tomography
CAA: cerebral amyloid angiopathy
GBM: glioblastoma
MCAO: middle cerebral artery occlusion
GnRH: gonadotropin-releasing hormone
IPP: Image-Pro Plus
DTI: diffusion tensor imaging
hMRI: histology using magnetic resonance imaging
IHC: immunohistochemical
mPFC: medial prefrontal cortex
Or: orbital area
Mo: motor area
Ac: anterior cingulate area
Ep: endopiriform nucleus
Cl: claustrum
Vi: visual area
Ss: somatosensory area
BST: bed nucleus of stria terminalis
MPO: medial pre-optic nucleus
WT: wild type
DPI: days post-injection
SINV: Sindbis virus
NF: neurofilament
TAG-1: transient axonal glycoprotein 1
CS: Carnegie stage
GW: gestational weeks
SCGN: secretagogin
